# Questionnaire on efficacy of the competency-oriented integrated residency and fellowship training for ophthalmologists in Shanghai

**DOI:** 10.3389/fmed.2026.1832809

**Published:** 2026-05-22

**Authors:** Shijia Li, Zichen Zhou, Manman Zhu, Minghui Zhao, Mengyang Gu, Ping Wang, Shiwei Li, Chang Liu, Da Long, Yan Chen

**Affiliations:** 1 Department of Ophthalmology, Shanghai Jiao Tong University Affiliated Sixth People’s Hospital, Shanghai, China; 2 Department of Computer Science, University of Warwick, Coventry, United Kingdom; 3 Department of Ophthalmology, Shanghai Municipal Hospital of Traditional Chinese Medicine, Shanghai University of Traditional Chinese Medicine, Shanghai, China

**Keywords:** competency, integrated residency and fellowship training, medical education, ophthalmologists, questionnaire

## Abstract

**Background:**

The integrated resident and fellowship (IRF) training represents a pivotal direction in China’s medical education reform, however, there are few reports detailing its content and efficacy. At the same time, the China’s six core competency concepts constitute the central objectives of this IRF training approach. Nevertheless, reports on the effectiveness of these six core competencies within the context of Shanghai’s IRF training implementation remain absent.

**Methods:**

The authors compared the competencies of medical education among China, Canda and the United States, and briefly reviewed the content of the integrated residency and fellowship training program for ophthalmologists in Shanghai. Furthermore, A survey using online questionnaire system was conducted to investigate the importance, implementation status, and existing deficiencies of the IRF training program for ophthalmologists in Shanghai among training instructors and resident or fellowship trainees.

**Results:**

Sixty-four valid questionnaires were collected with 30 instructors and 34 trainees. Regarding the importance of the training, more than 90% of the instructors and the trainees rated the six importance as “satisfactory” or better, with no statistically significant difference between the two groups (*p* > 0.05). Concerning the implementation status of training measures, 100% of both the instructors and the trainees rated “The training system is put into practice” as “satisfactory” or better. Additionally, 100% of the instructors and 89.2% of the trainees rated “truly achieving equal pay for equal work” as “satisfactory” or better, with no statistically significant difference between the two groups (*p* > 0.05). Regarding existing deficiencies, over 60% of both instructors and trainees indicated that “the supervision mechanism requires further enhancement,” with no statistically significant difference between the two groups (*p* > 0.05).

**Conclusion:**

Both instructors and trainees demonstrated a high level of recognition for the importance of the IRF training program for ophthalmologists in Shanghai. The implementation of measures also garnered widespread approval from both training instructors and trainees. However, further improvements are necessary to strengthen the supervision measures of the training program.

## 1 Introduction

Shanghai introduced standardized training for medical residents in 2010 and commenced fellowship training in 2013 (1). In 2015, nationwide standardized training for fellowships was officially launched (2). Since then, fellowship training in China has progressively evolved and explored integrated planning with resident training (3, 4).

Standardized trainings for resident and fellowship are pivotal stages in China’s post-graduation medical education. Traditionally, these two training programs have been relatively independent, each has its own distinct training objectives, curricula, and assessment criteria. However, with the rapid development of medical science and evolving demands for medical and healthcare services, this segmented training approach has gradually showed some deficiency such as inefficient resource utilization and poor continuity in training content (5–8). To optimize the pathway for cultivating medical professionals, the concept of integrated resident and fellowship (IRF) training model has emerged (9–12). This integrated model seeks to amalgamate the resources and curricula of both stages, ensuring a seamless transition between them and offering a more effective route for nurturing high-caliber fellowship physicians. As the IRF training gains promotion, Shanghai officially launched standardized fellowship training in 2024, prioritizing the IRF training as a key priority (13).

The medical education model in China adheres to a 5 + 3 + X framework: comprising 5 years of undergraduate medical study, 3 years of standardized resident training, and X years of fellowship physician training, where the duration of the X-year fellowship training ranges from 1 to 4 years, contingent upon the specific medical specialty and personal situation of the trainee doctors (2, 14, 15). In Shanghai, fellowship training typically spans 3 years. The objective of Shanghai’s IRF training system is to cultivate medical doctors endowed with excellent communication skills, effective teamwork capabilities, outstanding organizational, solid scientific research and educational aptitudes, independent clinical care competencies, and exceptional professional. These objectives are aligned with China’s six core competencies of doctors (16).

The six core competencies were introduced by the Accreditation Council for Graduate Medical Education (ACGME) and the American Board of Medical Specialties (ABMS) in 1999. The courses design, implementation, evaluation, assessment, and certification of physician training programs are all guided by these six core competencies (17). Resident physician training in Canada boasts a history spanning over a century, and its training system offers also valuable insights for optimizing China’s physician training framework, distinguished by its emphasis on seven role-based competency objectives (18). Similarly, China has also adopted six core competencies as orientation, with the objective of nurturing professionals who possess exemplary professional ethics, robust professional capabilities, and excel in coordinated patient management as well as communication and collaboration skills (16).

The IRF training represents a crucial direction in medical education reform in China. Nevertheless, its implementation encounters challenges including the formulation of training standards, faculty team construction, and the enhancement of assessment and evaluation systems. In this paper, the authors outlined the content of Shanghai’s IRF training program, along with the six core competencies. Utilizing a questionnaire survey, the authors investigated the effectiveness and insufficiency of Shanghai’s competencies-oriented IRF training for ophthalmologists, thereby contributing to the advancement of medical education.

## 2 Materials and methods

### 2.1 Study design

This study employed questionnaire survey methodology. The self-devised questionnaire was finalized after two rounds of deliberations with six experts from the Ophthalmology Department of the Sixth People’s Hospital, affiliated with Shanghai Jiao Tong University School of Medicine. The formulation of the questionnaire was based on the requirements outlined in national and Shanghai municipal policies pertaining to resident and fellowship training, the established evaluation criteria for such training programs, and an extensive review of relevant literature (19–21). The survey encompassed inquiries into the importance of IRF training, the implemented measures and their effectiveness, as well as the identified insufficiency.

### 2.2 Participants

The participants for the questionnaire survey in this study were residency and fellowship of ophthalmologists undergoing training, along with their training instructors in Shanghai. The survey was facilitated by the Shanghai Ophthalmologists Group and was conducted in October 2025 using an electronic questionnaire format (Wenjuanxing.wjx.cn. Changsha Ranxing Information Technology Co., Ltd., Changsha, China). The survey was conducted in the form of an electronic questionnaire in October 2025, with the online questionnaire collection period from October 1st to October 31st. The study was conducted in accordance with the local legislation, institutional requirements, and the ethical principles of the Declaration of Helsinki. Respondents scanned the questionnaire anonymously and continued to answer after scanning was deemed as consent. The survey is anonymous, and no personal information from respondents was collected, which adhered to the ethical principles of medical research.

### 2.3 Questionnaire survey

The questionnaire was reviewed by two ophthalmology teaching experts (Content Validity Index, CVI = 0.93). Before the official distribution of the questionnaire, a pre-test was conducted and tested its Cronbach’s *α* coefficient, which was α = 0.86, indicating that the questionnaire had high reliability and stability (22–24). The questionnaire was designed using a five-point Likert scale (1 = not helpful, 2 = general, 3 = helpful, 4 = very helpful, to 5 = extremely helpful) to assess subjective satisfaction with the IRF training for ophthalmologists in Shanghai. The questionnaires that are fully completed and devoid of logical errors are deemed valid.

### 2.4 Statistical analysis

All data were analyzed using SPSS version 25.0 (IBM Corp., Armonk, NY, USA). The Shapiro–Wilk test was first used to assess the normality of continuous variables. Normally distributed continuous data were expressed as mean ± standard deviation (x̄±s), Categorical variables were presented as percentages (%), and comparisons between instructors and trainees were conducted using the chi-square test. All statistical tests were two-sided, and an error level (*p*-value) < 0.05 was considered statistically significant.

## 3 Results

### 3.1 Description of instructors and trainees

A total of 64 valid questionnaires were collected in this survey. Among the respondents, there were 30 instructors, with 4 males and 26 females, and mean age 29 ± 6 years. In addition, there were 30 trainees, with 4 males and 30 females, and mean age 47 ± 13 years. Most participants held postgraduate degrees or higher, with only a very small number holding college or junior college degrees (Table 1).

**Table 1:** Basic information of instructors and trainees.

Participant	Age (years)	Sex		Degree		
		Male	Female	Junior college	College	Postgraduate or higher
Trainee	29 ± 6	4 (11.8%)	30 (88.2%)	0	2 (5.9%)	32 (94.1%)
Instructor	47 ± 13	4 (13.4%)	26 (86.6%)	2 (6.7%)	2 (6.7%)	26 (86.6%)

### 3.2 Importance of training

The questionnaire assessed instructors’ satisfaction with six key aspects of the IRF training program for ophthalmologists in Shanghai: improving trainees’ clinical skills, shortening the clinical learning curve, integrating high-quality resources from multiple hospitals, implementing hierarchical training management, establishing a diversified teaching evaluation system, and improving the overall training system.

For these six aspects, the proportion of instructors who rated them as “extremely helpful” was 66.7% (*n* = 20), 66.6% (*n* = 20), 60.0% (*n* = 18), 53.3% (*n* = 16), 46.7% (*n* = 14), and 46.6% (*n* = 14), respectively. The proportion rating them as “very helpful” was 33.3% (*n* = 10), 26.7% (*n* = 8), 33.3% (*n* = 10), 46.7% (*n* = 14), 40.0% (*n* = 12), and 26.7% (*n* = 8), respectively. Meanwhile, “helpful” responses accounted for 0, 6.7% (*n* = 2), 6.7% (*n* = 2), 0, 13.3% (*n* = 4), and 26.7% (*n* = 8), respectively.

**Figure 1:**
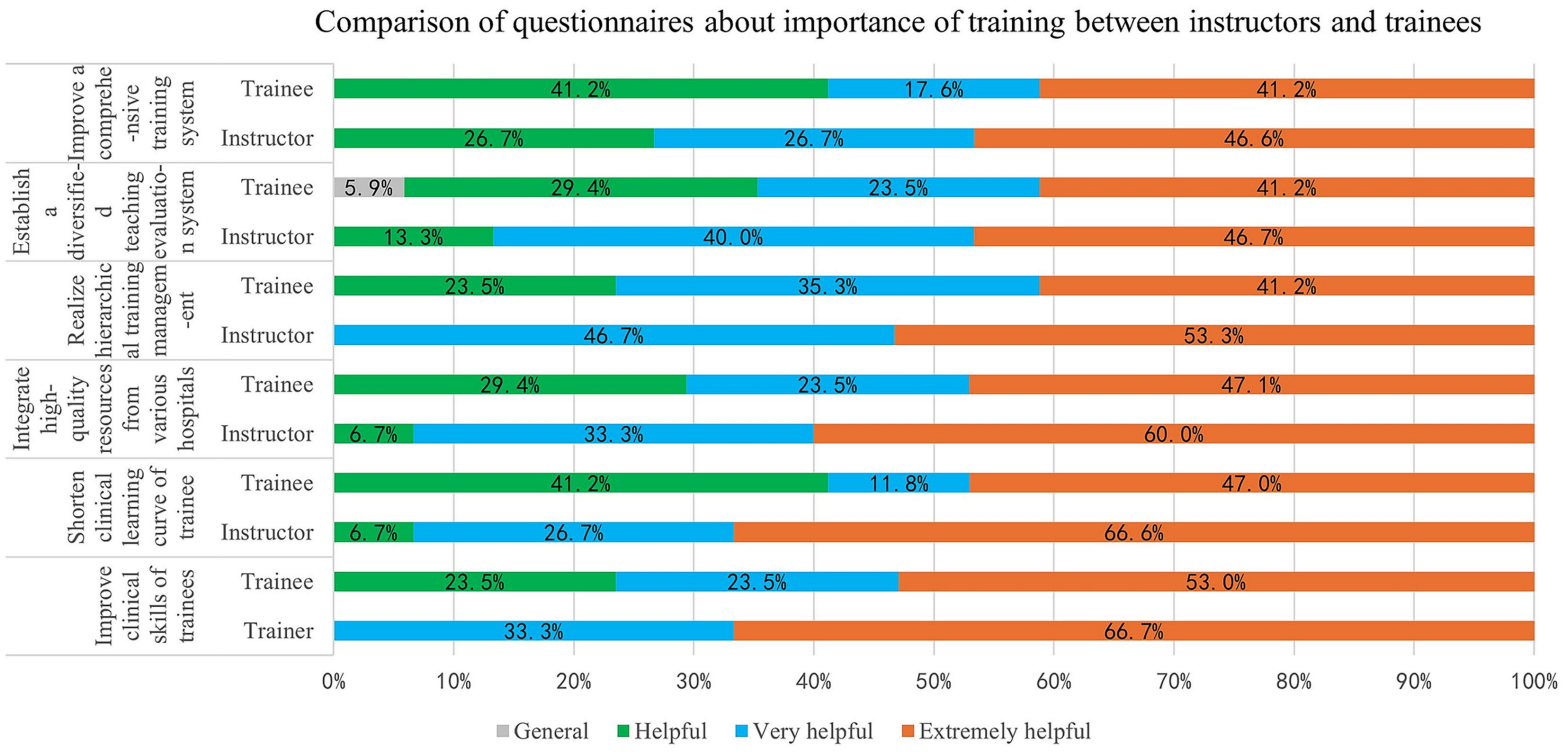
Questionnaire results of the main importance of training: Regarding “Improve clinical skills of trainees”, “Shorten clinical learning curve of trainee”, “Integrate high-quality resources from various hospitals”, “Realize hierarchical training management”, and “Improve a comprehensive training system”,100% of instructors and trainees selected “helpful” or better. Furthermore, 100% of instructors and 94.1% of trainees selected “Establish a diversified teaching evaluation system” as “helpful” or better, and 5.9% of trainees selected this item as “General”. There are no statistically significant differences between the instructor group and the trainee group about the main importance of training (*p* = 0.05).

The questionnaire results from trainees followed a similar format across the six assessed aspects. The proportion of trainees who rated each aspect as “extremely helpful” was 53.0% (*n* = 18), 47.0% (*n* = 16), 47.1% (*n* = 16), 41.2% (*n* = 14), 41.2% (*n* = 14), and 41.2% (*n* = 14), respectively. Ratings of “very helpful” accounted for 23.5% (*n* = 8), 11.8% (*n* = 4), 23.5% (*n* = 8), 35.3% (*n* = 12), 23.5% (*n* = 8), and 17.6% (*n* = 6), respectively, while “helpful” responses accounted for 23.5% (*n* = 8), 41.2% (*n* = 14), 29.4% (*n* = 10), 23.5% (*n* = 8), 29.4% (*n* = 10), and 41.2% (*n* = 14), respectively. Notably, for the aspect “establishing a diversified teaching evaluation system,” 5.9% (*n* = 2) of trainees selected “general.” Overall, there were no statistically significant differences between instructors and trainees regarding the perceived importance of the training components (*p* > 0.05) (Figure 1).

### 3.3 Implementation of training

The questionnaire also evaluated satisfaction with six key implementations of the IRF training program: the reasonableness of the top-level design, implementation of the training system in practice, shortening of the clinical learning curve, achievement of hierarchical and progressive management, facilitation of cross-hospital and interdisciplinary integration, and realization of equal pay for equal work.

Among instructors, the proportion rating these implementations as “extremely helpful” was 26.7% (*n* = 8), 20.0% (*n* = 6), 26.7% (*n* = 8), 26.7% (*n* = 8), 26.7% (*n* = 8), and 46.6% (*n* = 14), respectively. Ratings of “very helpful” accounted for 40.0% (*n* = 12), 40.0% (*n* = 12), 53.3% (*n* = 16), 53.3% (*n* = 16), 40.0% (*n* = 12), and 6.7% (*n* = 2), respectively, while “helpful” responses accounted for 26.7% (*n* = 8), 40.0% (*n* = 12), 20.0% (*n* = 6), 20.0% (*n* = 6), 33.3% (*n* = 10), and 26.7% (*n* = 8), respectively. Additionally, for the item “the top-level design is reasonable,” 6.6% (*n* = 2) of instructors selected “general.”

**Figure 2:**
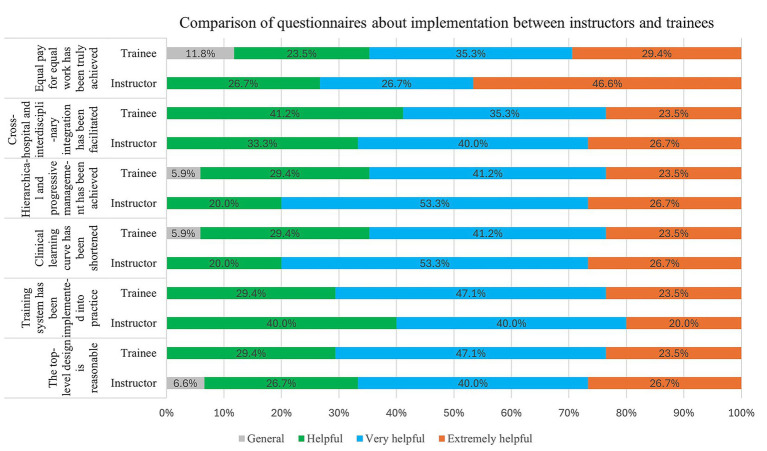
Questionnaire results of the implementations of training: Regarding “Training system has been implemented into practice”, and “Cross-hospital and interdisciplinary integration have been facilitated”, 100% of instructors and trainees selected “helpful” or better. Furthermore, 100% of instructors and 94.1% of trainees selected “Clinical learning curve has been shortened” and“Hierarchical and progressive management has been achieved” as “helpful” or better;100% of instructors and 88.2% of trainees have selected “Equal pay for equal work has been truly achieved” as “helpful” or better; and 93.4% of instructors and 100% of trainees have selected “The top-level design is reasonable” as “helpful” or better. There are no statistically significant differences between the instructor group and the trainee group about the implementations of training (*p* > 0.05).

Among trainees, the proportion rate of the implementations as “extremely helpful” was 23.5% (*n* = 8), 23.5% (*n* = 8), 23.5% (*n* = 8), 23.5% (*n* = 8), 23.5% (*n* = 8), and 29.4% (*n* = 10), respectively. Ratings of “very helpful” were 47.1% (*n* = 16), 47.1% (*n* = 16), 41.2% (*n* = 14), 41.2% (*n* = 14), 35.3% (*n* = 12), and 35.3% (*n* = 12), respectively, and “helpful” responses were 29.4% (*n* = 10), 29.4% (*n* = 10), 29.4% (*n* = 10), 29.4% (*n* = 10), 41.2% (*n* = 14), and 23.5% (*n* = 8), respectively. Notably, 5.9% (*n* = 2) of trainees selected “general” for both “clinical learning curve has been shortened” and “hierarchical and progressive management has been achieved,” while 11.8% (*n* = 4) selected “general” for “equal pay for equal work has been truly achieved.” Overall, there were no statistically significant differences between instructors and trainees regarding satisfaction with the implementation of the training program (*p* > 0.05) (Figure 2).

### 3.4 Insufficiency of training

A majority of respondents identified several key limitations of the training program. Specifically, 60.0% (*n* = 18) of instructors and 70.6% (*n* = 24) of trainees indicated that supervisory measures require further implementation to better safeguard trainees’ interests. The same proportions also noted that, due to limited equipment across hospitals, only short-term cross-hospital training can currently serve as a compensatory measure.

In addition, 66.7% (*n* = 20) of instructors and 47.1% (*n* = 16) of trainees reported that limited faculty resources hinder the full realization of predetermined goals and strategies. Concerning the need for further refinement and systematization of the training system were raised by 53.3% (*n* = 16) of instructors and 41.2% (*n* = 14) of trainees. Similarly, 53.3% (*n* = 16) of instructors and 35.3% (*n* = 12) of trainees identified a lack of motivation or burnout among trainees as an issue.

**Figure 3:**
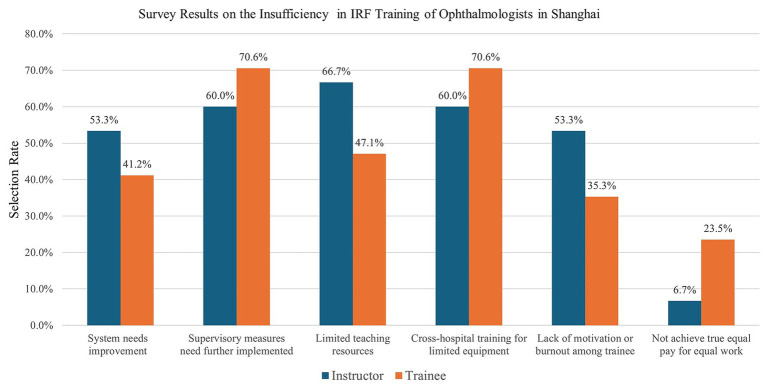
Questionnaire Survey results about the insufficiency of the training: 60% of instructors and 70.6% of trainees selected the options indicating that “The supervisory measures necessitate further implementation to safeguard the interests of the trainee.” and that “Due to the limited equipment across different hospitals, only short-term cross-hospital training can serve as a compensatory measure.” Merely 6.7% of instructors and 23.5%of trainees selected the options indicating that “True equal pay for equal work has not been attained.” There are no statistically significant differences between the instructor group and the trainee group about the insufficiency of the training (*p* = 0.05).

By contrast, relatively few respondents indicated that true equal pay for equal work has not been achieved, with only 6.7% (*n* = 2) of instructors and 23.5% (*n* = 8) of trainees selecting this option. Overall, there were no statistically significant differences between instructors and trainees regarding perceived shortcomings of the training program (*p* > 0.05) (Figure 3).

## 4 Discussion

This study described the similarities and differences in competency descriptions across three teaching models from the United States, Canada, and China (Table 2). It is evident that the core competencies in all three countries encompass the enhancement of medical knowledge, communication and collaboration skills, and patient health management. The difference lies in the that the six core competencies in the United States specifically include medical knowledge and practical skills as two distinct domains, whereas in China’s six core competencies, the elements of medical knowledge and practical skills are consolidated into professional competence, with the inclusion of teaching ability. Conversely, Canada’s seven core competencies incorporate leadership as an additional component (16–18). Both teaching ability and leadership assume pivotal roles in a doctor’s professional career (25, 26)^.^ If the cultivation of these competencies is fully actualized, it will undoubtedly make a substantial contribution to the comprehensive enhancement of the trainees’ capabilities (27–30).

**Table 2:** Similarities and differences in competencies importance among China, the United States, and Canada.

America	China	Canada
Medical knowledge	Expertise	Medical expert
Patient care	Patient management	Communicator
Interpersonal and communication skills	Communication and collaboration	Collaborator
Practice-based learning improvement	Learning enhancement	Leader
Professionalism	Professionalism	Health advocate
Systems-based practice	Teaching ability	Scholar
		Professional

The IRF training program for ophthalmologists in Shanghai consists of two sequential phases: a three-year standardized resident training phase followed by a three-year standardized fellowship training phase. The relationship between these two phases adheres to a tiered progression model. The primary training components of the resident training phase encompass: (1) Professional competency development for residents, which includes independent acquisition and documentation of ophthalmic medical histories, autonomous conduct of ophthalmic examinations and formulation of ophthalmic treatment strategies, performance of corneal suture surgeries on animal eyes, and participation in fundamental ophthalmic surgical procedures; (2) Training in communication and collaborative skills for residents, covering doctor-patient communication proficiency and interdepartmental communication and coordination. The core training elements of the fellowship training phase involve: (1) Basic clinical skills training during the first and second years, focusing on achieving mastery in diagnosing and managing a wide array of common and complex ophthalmic conditions, as well as acquiring proficiency in ophthalmic surgical techniques such as rectus muscle surgeries and phacoemulsification; (2) Advanced clinical skills training in the third year, wherein each fellowship selects a subspecialty to deepen their expertise in diagnosing and treating clinical diseases specific to that subspecialty and to refine their surgical techniques accordingly (Tables 3, 4) (19–21). This IRF training framework encompasses the enhancement of professional skills, communication and teaching abilities, and patient management, aligning clearly with China’s six core competencies (16).

**Table 3:** Professional competency training for ophthalmologists at residency stage of integrated residency and fellowship model.

Professional competency training for ophthalmologists at residency stage of residency-specialty-integration model								
The first year	Value	Score	The second year	Value	Score	The third year	Value	Score
Participate in popular science propaganda	10		Participate in popular science lecture	15		Educate patients on disease prevention and first aid knowledge	10	
Patient health education	20		Patient health education	15		First aid knowledge lecture in community	20	
Small lecture in class	15		Main lecturer of specialized lectures in the department	15		National academic conference exchange	15	
Literature presentation	15		Translate professional literature	15		Participate in research projects at or above the institute level	15	
Professional competence evaluation for serving patients -evaluation by teaching staff	10		Professionalism in the medical behavior process—evaluation by teaching staff	10		Provide high-quality professional services to patients	10	
Professional competence evaluation for serving patients—evaluation by nurses	10		Professionalism in the medical behavior process—evaluation by nurses	10		Good medical ethics and conduct, no complaints	10	
Professional competence evaluation for serving patients—evaluation by patients’ Families	10		Professionalism in the medical behavior process—evaluation by patients’ Families	10		Received praise from doctors, nurses or from patients and their families	10	

**Table 4:** Communication and collaboration skills training for ophthalmologists at residency stage of integrated residency and fellowship model.

Communication and collaboration skills training for ophthalmologists at residency stage of residency-specialty-integration model								
The first year	Value	Score	The second year	Value	Score	The third year	Value	Score
Participate in entrance education	5		Proficient in doctor-patient communication	10		Effectively communicate with all departments	10	
Good communication	5		Communicate diagnosis and treatment plans, and no errors or accidents	10		Assist in referring patients	10	
Independently collect ophthalmic medical history	10		Guide communication skills for junior doctors, same level doctors, and nurses	10		Community or medical consortium work	10	
Assist in making ophthalmic diagnosis and treatment plans	10		Assist in handling clinical issues	10		Participate in interdisciplinary professional consultation	10	
Report meaningful clinical manifestations	10		Participate in the rescue of ophthalmic emergency patients	10		Join medical team	10	
Collaborate solving problem	10		Guide interns and trainees in their work	10		Participate in community publicity	10	
Job Responsibilities	10		Relatively good allocation of medical learning group resources	10		Participate in management of medical resources within department	10	
Hygiene regulations	10		Borrowing resources from other medical study groups	10		Assist the chief resident in managing work	10	
Rules and regulations	10		Assist superiors in teaching management	10		Participate in individualized management of critically ill patients	10	
Participate in training activities	10							

As previously mentioned, the “six core competencies” serve as the principal objectives for postgraduate medical education in China. The IRF training program for ophthalmologists in Shanghai is also designed around these “six core competencies.” Its aim is to significantly elevate the capabilities of medical students by optimizing a comprehensive teaching management system and teaching assessment framework, along with adopting a tiered and progressive training model (31–33). The endorsement and positive feedback from instructors and trainees indicate that these importances of the IRF training program have been broadly acknowledged and accepted by both instructors and trainees. These outcomes could lay a robust personnel foundation and offer strong subjective impetus for seamless execution of the IRF training program.

Focusing on the six core competencies, the principal measures currently adopted in the IRF training program for ophthalmologists in Shanghai include: (1) The implementation of a stratified and phased training and assessment management framework to continually deepen the clinical skills development of trainees; (2) The establishment of a mentorship accountability system, where both resident and fellowship trainees receive personalized guidance from experienced mentors on an individual basis; (3) For subspecialties not offered in certain training hospitals, trainees are provided with the opportunity to pursue studies at hospitals with relevant specialized expertise, thereby promoting resource sharing; (4) The utilization of a comprehensive examination and evaluation system, in conjunction with a bidirectional feedback mechanism between trainees and mentors, to enhance the monitoring and evaluation of training efficacy; (5) The enforcement of equal pay for equal work within training hospitals to ensure that trainees have sufficient financial means to support their living expenses (19–21).

The questionnaire survey results demonstrate that the instructors and trainees express a relatively high level of satisfaction with the implementation pertaining to the IRF training program for ophthalmologists in Shanghai. 100% of instructors and trainees have selected the “Training system has been implemented into practice” as helpful or better. Furthermore, 100% of instructors and 88.2% of trainees have selected “Equal pay for equal work has been truly achieved” as helpful or better. Equal pay for equal work stands as one of the hallmarks of doctor training in Shanghai, providing a significant economic safeguard for the learning and daily lives of trainees. In comparison with the importance, the proportion of “Extremely helpful” with the implementation of the measures has markedly declined.

The survey results concerning the implementation of Shanghai’s IRF training measures in ophthalmology highlight the city’s formidable execution capability in physician training. Simultaneously, it is imperative to acknowledge that, when compared to relatively mature countries in resident-fellowship training, such as the United States (34–36), Canada (37, 38), and Shanghai’s training strategies, particularly those aimed at enhancing the six core competencies of physicians, still require further enhancement (39–41).

The results of this questionnaire demonstrate that over 60% of instructors and trainees have opted for the statement, “The supervisory measures necessitate further implementation to safeguard the interests of the trainees.” The configuration of supervisory bodies for postgraduate medical education in China is continually being optimized, incorporating institutions such as the Health Commission, the Municipal Education Commission, the Ophthalmology Branch of the Chinese Medical Doctor Association, and the graduation education offices within hospitals. The supervision of postgraduate medical education is derived from the concerted efforts of these institutions. Additionally, the supervisory and feedback roles played by instructors and trainees constitute essential elements of the supervisory mechanism (41, 42).

A substantial proportion of instructors and trainees have selected “the limited equipment across different hospitals can only be compensated by cross-hospital training programs.” Given the disparities in resource variations among hospitals, trainees in specialized training are afforded the opportunity to engage in cross-hospital training across major hospitals in Shanghai. This situation not only underscores a deficiency in the IRF training system in Shanghai but also accentuates the collaborative strength and unity among the city’s leading hospitals. As resources of hospital continue to augment, these issues are anticipated to be effectively resolved.

### 4.1 Strengths and limitations

Strengths: The authors employed questionnaires to compare and analyze the implementation status of IRF training oriented toward China’s six core competencies in Shanghai from the viewpoints of both training instructors and trainees, thereby providing valuable insights for further research and refinement on this post-graduation education system. As far as we are aware, this marks the inaugural report on research pertaining to the IRF training in Shanghai, especially the research on the IRF oriented toward China’s six core competencies. Limitations: This article offered a cross-sectional depiction of IRF oriented toward China’s six core competencies in Shanghai through questionnaires. Further prospective cohort studies and in-depth analysis should be conducted to improve the IRF training frameworks, with an emphasis on enhancing the China’s six core competencies of training physicians.

## 5 Conclusion

The integrated residency and fellowship training system for ophthalmologists in Shanghai is guided by six core competencies and adopts a stratified, progressive training model. Its primary aim is to ensure systematic and all-encompassing enhancement of the competencies of trainee doctors through the seamless integration of the residency and fellowship training stages. The results of this survey questionnaire demonstrate that both instructors and trainees exhibit a high level of endorsement for the importance of Shanghai’s integrated residency and fellowship training system in ophthalmology. The implementation of the training measures has also received widespread approval from instructors and trainees, with satisfaction expressed regarding equal pay for equal work. However, further improvements are required in areas such as strengthening the training supervision measures.

## Data Availability

The original contributions presented in the study are included in the article/supplementary material, further inquiries can be directed to the corresponding authors.
